# A highway vehicle routing dataset during the 2019 Kincade Fire evacuation

**DOI:** 10.1038/s41597-022-01731-6

**Published:** 2022-10-07

**Authors:** Yiming Xu, Xilei Zhao, Ruggiero Lovreglio, Erica Kuligowski, Daniel Nilsson, Thomas J. Cova, Xiang Yan

**Affiliations:** 1 University of Florida, Department of Civil and Coastal Engineering, Gainesville, Florida 32611 USA; 2 Massey University, School of Built Environment, Auckland, 0632 New Zealand; 3 RMIT University, School of Engineering, Melbourne, Victoria 3000 Australia; 4 University of Canterbury, Department of Civil and Natural Resources Engineering, Christchurch, 8041 New Zealand; 5 University of Utah, Department of Geography, Salt Lake City, Utah 84112 USA

**Keywords:** Geography, Research data, Social sciences

## Abstract

As the threat of wildfire increases, it is imperative to enhance the understanding of household evacuation behavior and movements. Mobile GPS data provide a unique opportunity for studying evacuation routing behavior with high ecological validity, but there are little publicly available data. We generated a highway vehicle routing dataset derived from GPS trajectories generated by mobile devices (e.g., smartphones) in Sonoma County, California during the 2019 Kincade Fire that started on October 23, 2019. This dataset contains 21,160 highway vehicle routing records within Sonoma County from October 16, 2019 to November 13, 2019. The quality of the dataset is validated by checking trajectories and average travel speeds. The potential use of this dataset lies in analyzing and modeling evacuee route choice behavior, estimating traffic conditions during the evacuation, and validating wildfire evacuation simulation models.

## Abstract (summary)

**Table:** 

Measurement(s)	highway vehicle routing
Technology Type(s)	GPS location tracking
Sample Characteristic - Location	Sonoma County, California, USA

## Background & Summary

The intensity and frequency of wildfires continues to grow^1–6^. For instance, the 2020 California, Oregon, and Washington Firestorms burned over five million acres and destroyed thousands of buildings, causing over 500,000 people to evacuate and two dozen fatalities^7^. To enhance emergency response and public safety, it is imperative to expand the understanding of household evacuation behavior and movements in wildfires. Such knowledge can help authorities develop appropriate emergency response plans and make effective decisions during a wildfire event. This includes planning traffic management strategies, issuing evacuation orders, providing support for disadvantaged travelers, and undertaking rescues^8,9^.

Capturing evacuees’ routing behaviors is important to estimate traffic conditions, identify bottlenecks, and develop real-time corresponding traffic control strategies. However, there is limited research on this topic due to a lack of detailed publicly-available data. Existing disaster response research focuses on the evacuation decision (whether to evacuate or stay) using data sources such as surveys and interviews^3,5,8–10^. Although these data have detailed individual-level information to provide a fundamental understanding of the wildfire evacuation decisions, they have limited information on evacuee movements (i.e., routing behavior) during the evacuation. Additionally, as these traditional data rely on people’s memory, it is difficult to collect accurate timestamps and locations of their evacuation routes, which introduces challenges in understanding their movements and estimating traffic conditions during evacuation. The mobile GPS data, which are location data records generated by capturing the satellite pings that are transmitted through mobile device applications, provide a unique opportunity to complement the data collected using questionnaires and to enhance our understanding of people’s evacuation routing behavior by providing highly granular spatiotemporal movement information. Compared with survey methods, a GPS dataset is collected automatically by a mobile device, has a large sample size (e.g., millions of observations), and provides approximate timestamps and GPS locations of people’s movements. These characteristics make GPS data an appropriate data source for capturing people’s routing behavior during the evacuation, which can be used to validate existing evacuation simulation models^11,12^. However, *there is no publicly available GPS dataset to allow researchers and practitioners to analyze people’s routing behavior during wildfire evacuations*.

**Fig. 1:**
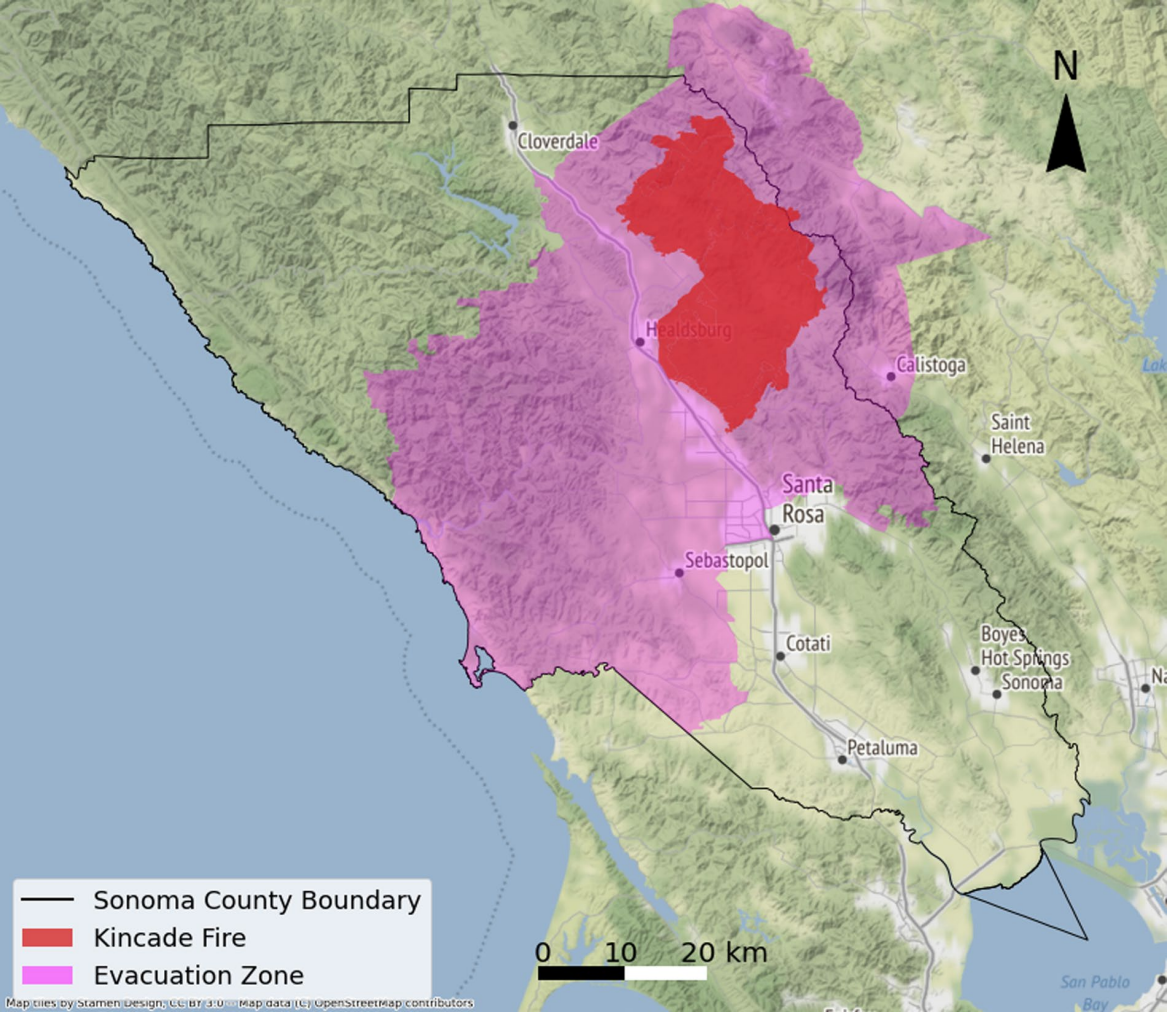
Sonoma County and the Kincade Fire Perimeter.

In this study, we provide a highway vehicle routing dataset derived from the GPS trajectories in Sonoma County, California that represents people’s movements during the 2019 Kincade Fire. The Kincade Fire started in Sonoma County at 9:27 p.m. on October 23, 2019 and was fully contained at 7:00 p.m. on November 6, 2019. The fire burned 77,758 acres, destroyed 374 structures, damaged 60 structures, and caused 4 injuries^13^. As the fire spread, a mandatory evacuation order was first issued on October 26, and then the evacuation warnings and orders grew to encompass most of Sonoma County in the following days. The study site, the fire perimeter, and the evacuation zones (indicating the geographic area under evacuation warnings/orders) are shown in Fig. 1.

This dataset can be used to study how households responded to wildfire evacuation warnings and orders and to explore the impacts of evacuation notifications on traffic flows. The potential use of this dataset includes: (1) analyzing and modeling evacuee route choice behavior; (2) estimating traffic conditions (e.g., travel time, traffic flow and speed); and (3) validating existing wildfire evacuation simulation models^11,12^.

## Methods

The GPS dataset (or, Observations data) was provided by Gravy Analytics^TM^ and built on privacy-friendly mobile location data. Gravy processes billions of raw mobile location signals each day to build Observations data–cleansing and deduping the data to eliminate fraudulent, problematic, and duplicate data. Gravy also applies Forensic Flags to the data to classify and filter valid data by signal origin, location accuracy, and other key characteristics. This separates high-quality signals from low-quality, suspicious, and even fraudulent signals and enables analysts to select and use only the data needed for their specific use case. Additionally, Gravy Analytics takes consumer privacy seriously and ensures that its data remains compliant with industry and legal requirements. Gravy is intimately familiar with the obligations established by the CCPA, GDPR, and other privacy laws around the world. Gravy works with its data suppliers to ensure the data the company processes comes from device users who have opted-in to the collection of device identifiers and geolocation signals. Gravy applies this standard on a global level, regardless of whether this requirement exists in the jurisdiction where the device is present. Gravy maintains a robust privacy request channel designed to satisfy the obligations established by the world’s privacy laws. Accordingly, the Gravy privacy team diligently responds and complies with each privacy request they receive in a timely manner. Users can submit their own opt-out or privacy request on Gravy’s website. For more information on how Gravy protects consumer privacy, see here: https://gravyanalytics.com/consumer-privacy/. If interested in obtaining the type of data used in this study, readers can reach out to Gravy Analytics via the contact form (https://gravyanalytics.com/contact-us/). Then, a Gravy representative will reach out to discuss the specific data request and additional details, such as payment, delivery date, etc.

To further preserve the privacy of the users and comply with the contractual requirements of the data provider, we only extracted data points reflecting a vehicle’s proxy entrance or exit of a particular highway(s) (i.e., the first or last record on the highway(s)). This dataset does not include records that can be used to deduce daily activity locations but can provide the vehicle routing information on highways before, during, and after the Kincade Fire. This dataset is anonymous, but it can provide valuable information for researchers and practitioners to investigate evacuation behavior of residents.

**Fig. 2:**
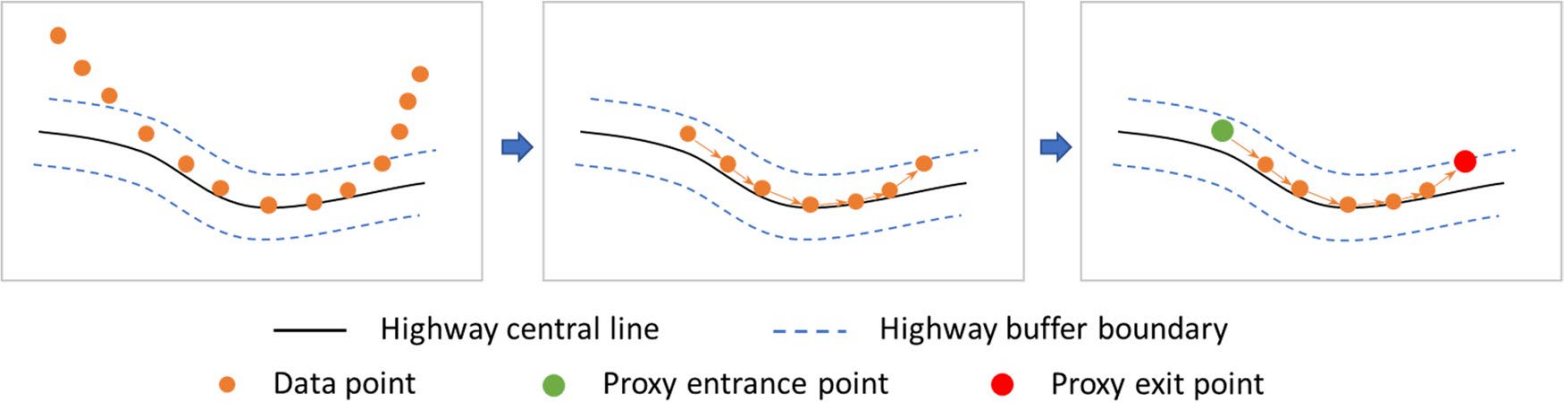
An example of data processing.

**Fig. 3:**
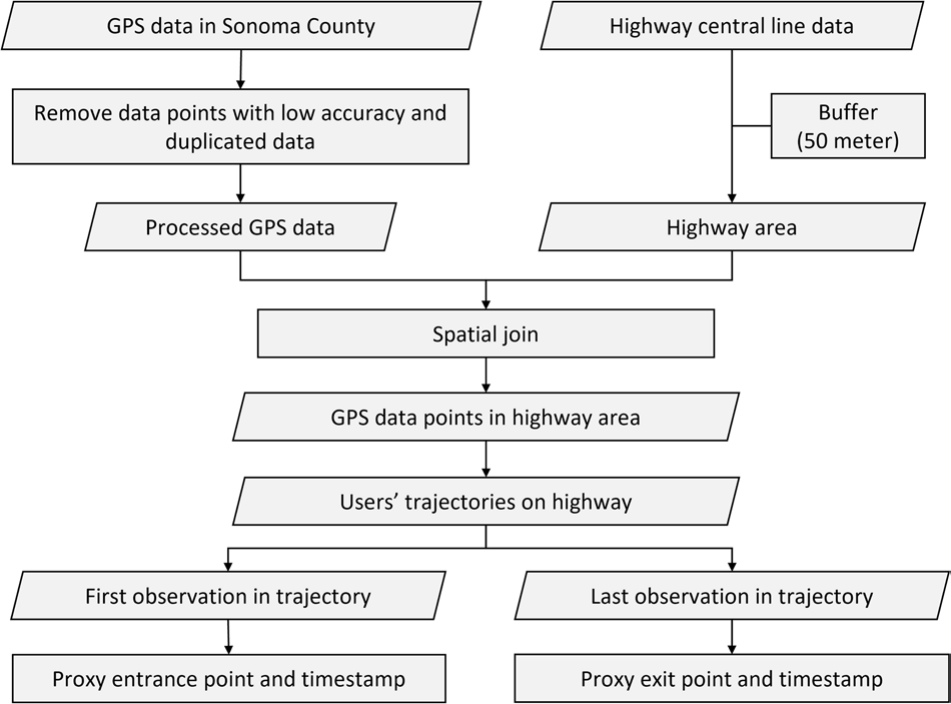
Schematic overview of the data processing method.

**Fig. 4:**
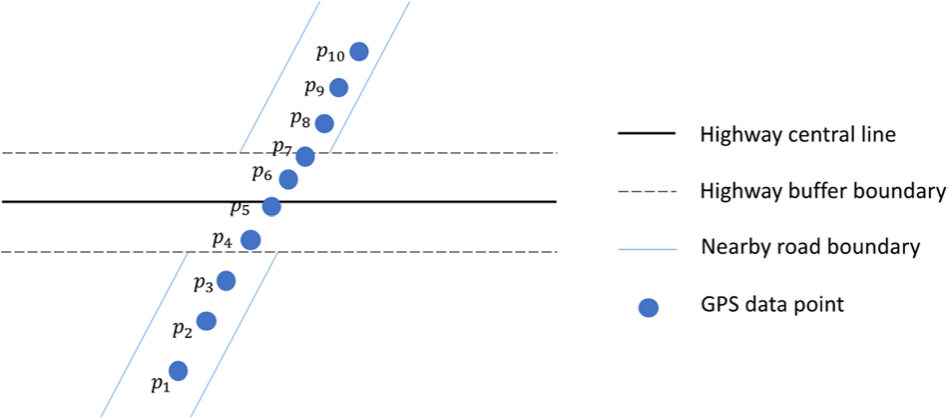
An example of interchange of highway and nearby road of other types.

The pre-processed GPS data includes records for mobile device users (we refer them as users in the following text for simplicity) in Sonoma County from one week prior to the Kincade Fire to one week after the fire (i.e., October 16, 2019 to November 13, 2019). Database fields of the GPS data include the unique identifiers for devices, geohash latitude, geohash longitude, the geohash (a geocode format using a short alphanumeric string to express a location, find more details here http://geohash.org/site/tips.html), timestamp, time zone, and a flag indicating the GPS accuracy. A synthetic sample of GPS data is presented in Table 1. To extract the highway vehicle routing data for publication, we removed the duplicate records for each unique identifier according to the locations and the timestamps. If an identifier had multiple records with the same locations (i.e., the same geohash) and timestamps (resolution is 1 minute), only one record was retained. We used GIS tools to conduct a spatial join for the GPS records and the highways (i.e., U.S. Highway 101, and State Highways 1, 12, 37, 116, 121, and 128) within Sonoma County (see Fig. 2). The highway center lines were buffered by *b* meters to represent the highway areas. According to California’s Highway Design Manual^14^, the overall width of an 8-lane (4 lanes for each direction) highway is approximately 42 meters. We thus assumed that the width of the highways is less than 50 meters, i.e., the buffer was set as *b* = 50 m. Based on the spatial join results, we extracted the GPS records located in the highway areas. We then joined these GPS records into trajectories based on the identifier and timestamp. We noticed that the algorithm may produce inaccurate trajectories in highway interchanges with roads of other types. For example, in Fig. 4, point *p*_4_ to *p*_7_ of a user’s trip (traveling from *p*_1_ to *p*_10_) may be identified as a highway trajectory. In this type of scenarios, the length of the identified highway trajectory will be very small. Therefore, we set a minimum length threshold for the identified highway trajectories to eliminate these inaccurate records. As prior work indicated that the recommended minimum distance between two successive ramps is 2500 ft (762 meters) for a highway with free flow speed of 65 mph^15^, we thus set the minimum length threshold for the identified highway trajectories to 2500 ft (762 meters). We also removed the duplicate trajectories with the same entrance and exit location but very small fraction difference in timestamp (less than 1 minute) as a result of multiple signals from the same GPS device. Based on the highway trajectories, we extracted the start and end points of these trajectories as the vehicles’ proxy entrance and exit points to highways. The data processing steps are presented in Fig. 3.

**Table 1:** Synthetic GPS Data Samples.

ID	LATITUDE	LONGITUDE	GEOHASH9	TIMESTAMP_EPOCH	TIMEZONE	FLAG
00001	*y*_1_	*x*_1_	9qbd*****	15715********	TZ1	0
00002	*y*_2_	*x*_2_	9qbc*****	15715********	TZ1	0
00003	*y*_3_	*x*_3_	9qbs*****	15712********	TZ1	0
00003	*y*_4_	*x*_4_	9qbe*****	15726********	TZ1	0
00004	*y*_5_	*x*_5_	9qbd*****	15713********	TZ1	0
00004	*y*_6_	*x*_6_	9qbd*****	15714********	TZ1	0

## Data Records

The highway vehicle routing dataset^16^ can be accessed from the DesignSafe-CI data portal at: 10.17603/ds2-9v8w-y830. This dataset contains vehicles’ routes on highways (within Sonoma County) from October 16, 2019 to November 13, 2019. The total number of records in this dataset is 21,160. Note that all data were included in this dataset. It is up to the data user to do the filtering. The dataset are provided in CSV (comma-separated values) format. The data fields include the anonymous identifier for the mobile device, the location (i.e., geohash latitude and geohash longitude) reflecting a vehicle’s proxy entrance or exit to a highway, the corresponding timestamp, and the corresponding highway name. The detailed descriptions of variables are presented in Table 2. We also provide a shapefile of the highways (i.e., U.S. Highway 101, State Highways 1, 12, 37, 116, 121, and 128) as a complementary file. Based on this dataset, the vehicle routing information can be directly obtained for further analysis.

**Table 2:** Description of Variables.

Variable	Description
ID	Anonymous identifier for mobile devices
Entrance_LAT	Latitude of vehicle’s proxy entrance of highway
Entrance_LON	Longitude of vehicle’s proxy entrance of highway
Entrance_TIMESTAMP	Timestamp of vehicle’s proxy entrance of highway as epoch time in milliseconds
Entrance_HWY	Entrance highway
Exit_LAT	Latitude of vehicle’s proxy exit of highway
Exit_LON	Longitude of vehicle’s proxy exit of highway
Exit_TIMESTAMP	Timestamp of vehicle’s proxy exit of highway as epoch time in milliseconds
Exit_HWY	Exit highway

## Technical Validation

The accuracy of GPS location records can influence the quality of the dataset^17,18^. Therefore, to ensure the quality of the highway vehicle routing dataset, we cleaned the GPS data by removing records with low GPS accuracy (i.e., >50 meters). The GPS accuracy is identified by the GPS accuracy flag named Forensic Flag in the GPS dataset. The Forensic Flag is generated by the GPS data provider, Gravy Analytics. We also eliminated duplicate observations in this process.

According to the data generation process described in *Methods* section, we first obtained the users’ trajectories on the highways and then outputted the first and last observation of each trajectory as the proxy entrance and exit point. In other words, for each record (i.e., row) in the highway vehicle routing dataset, the corresponding user was traveling on the highway between the entrance and exit data points.

**Fig. 5:**
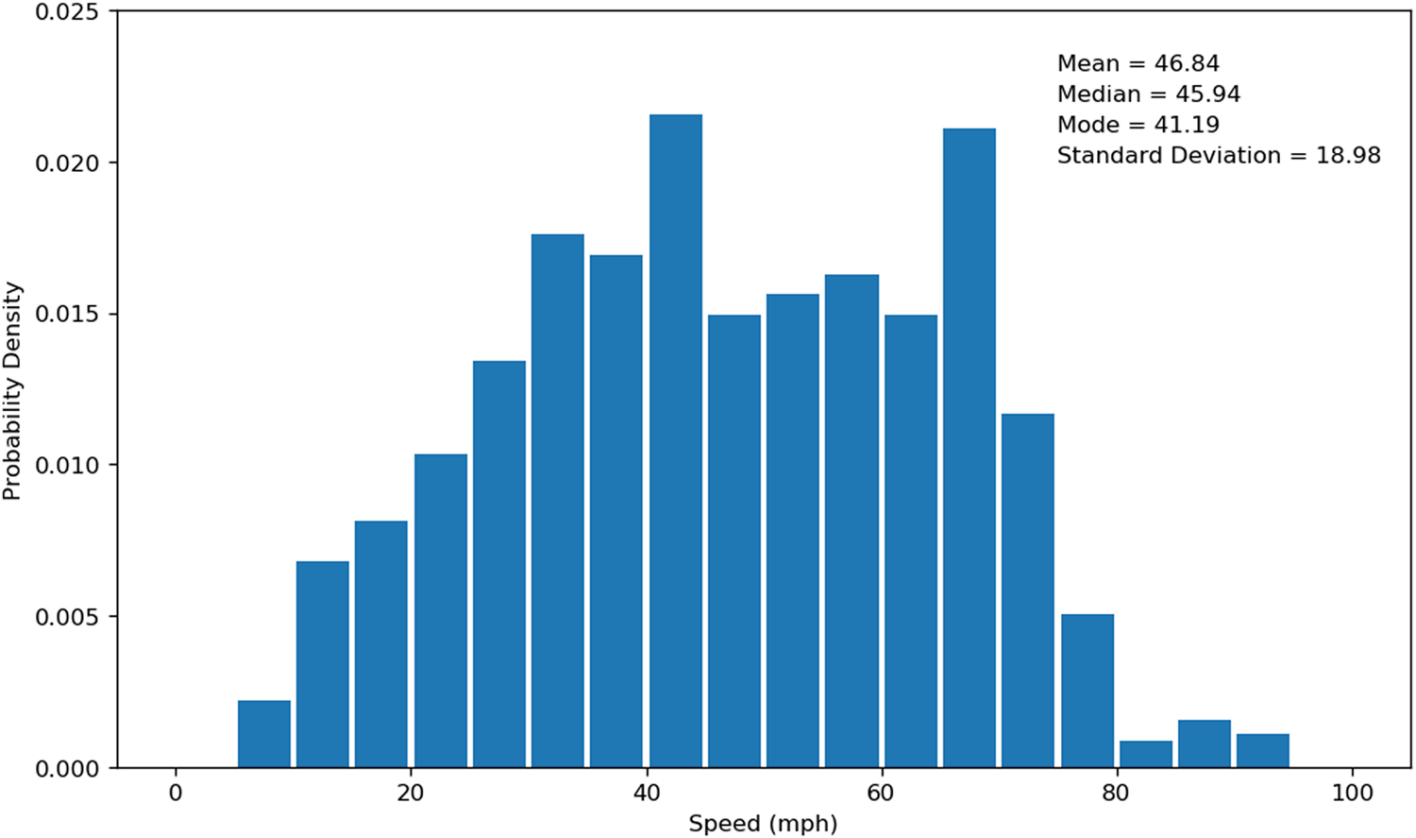
Travel speed distribution (before the fire started).

**Fig. 6:**
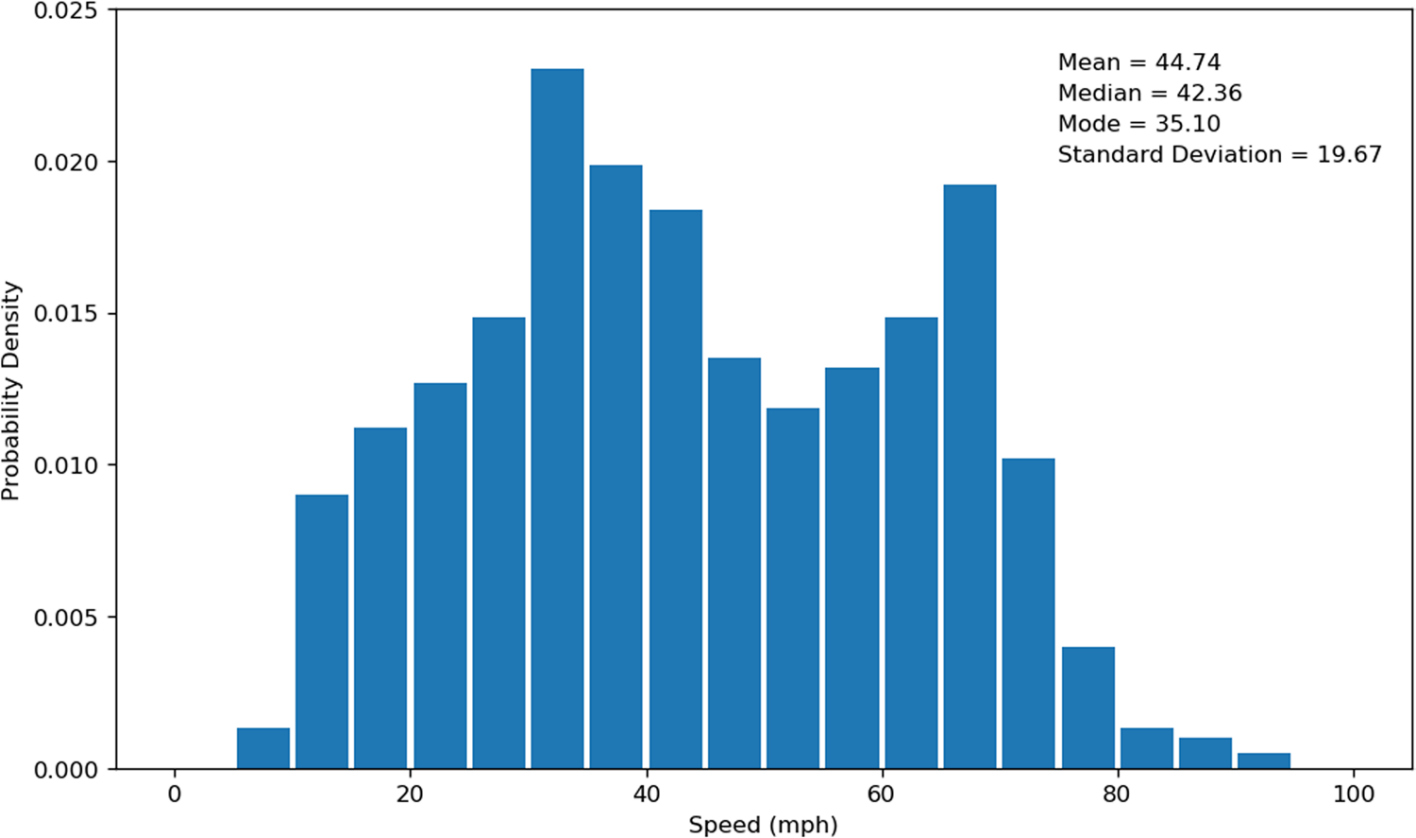
Travel speed distribution (during the fire).

We also used the distribution of the evacuee average travel speed for each trip to validate the highway vehicle routing dataset. The average travel speed was estimated by using the road network distance between the data points reflecting the user’s entrance and exit of the highway divided by the corresponding travel time. The distributions of average travel speed before the wildfire (i.e., Saturday, October 19, 2019 to Sunday, October 20, 2019) and during the wildfire (i.e., Saturday, October 26, 2019 to Sunday, October 27, 2019) are presented in Figs. 5 and 6. Note that most evacuations occurred on October 26–27, 2019 during the Kincade Fire^6^, so we selected these two dates to generate the travel speed distribution during the fire. As these two dates were weekend days, we then selected October 19–20, 2019 (the weekend before the fire started) to generate the travel speed distributions before the fire as a comparison. Before the fire started, the mean of mobile device users’ average travel speed is 46.84 mph, the median is 45.94 mph, the mode is 41.19 mph, and the standard deviation is 18.98 mph. During the fire, the mean of the average travel speed is 44.74 mph, the median is 42.36 mph, the mode is 35.10 mph, and the standard deviation is 19.67 mph. We can observe that the average travel speed during the wildfire evacuation is smaller than before the wildfire, and this result is consistent with previous studies^19,20^.

We then tested whether the travel speed distribution before the fire is significantly different from that during the fire. In this case, we used the Kolmogorov-Smirnov test (K-S test)^21,22^ to evaluate whether the two distributions are from the same dataset. The *P*-value achieved by the test provides insight on whether we can reject the hypothesis that two speed samples are drawn from the same distribution. We applied the two-sample K-S test to the average travel speed before and during the wildfire, and obtained the *P*-value = 0.0022, which is well below the commonly-used 0.05 threshold, suggesting that we have strong evidence to reject the null hypothesis. The results of K-S test indicate the distributions of average travel speed before and during the wildfire are significantly different, which is also consistent with previous studies^19,20^.

## Usage Notes

The highway vehicle routing dataset^16^ is distributed as a CSV file with data fields summarized in Table 2 and a shapefile of the highways in Sonoma County. Since the dataset was extracted from the GPS dataset with various time intervals, the entrances and exits revealed by the dataset may not be the exact locations of actually highway entrances (i.e., on-ramps) and exits (i.e., off-ramps). One may need to attach them to the nearest highway entrance/exit before using the data for analysis. In some cases, we have a vehicle’s proxy entrance and exit point across multiple highways, so one has to infer potential paths from one highway to another to estimate the probable travel distance.

The highway vehicle routing dataset^16^ is free for use/reuse. This dataset can be directly used to analyze and model people’s route choice before and during the emergency. This dataset can also be used to validate existing evacuation simulation tools, especially by examining the route choice behavior. Additionally, one can leverage this dataset to understand travel delays during wildfire evacuation. Since the dataset provides the location and the timestamp of vehicles proxy entrance and exit of the highway, one can estimate the travel time between two locations during the wildfire using this dataset. The estimated travel time can be compared with the regular travel time to estimate the travel delays during the wildfire evacuation. Using the travel time and the proxy entrance and exit to highway(s), one can also derive traffic speed on a specific highway segment to identify highway hot spots and bottlenecks during the wildfire evacuation. Moreover, this dataset can be used to understand the association between the traffic and the built environment (e.g., number of lanes, total combined width of all lanes, speed limit, urban area, etc.), to inform transportation infrastructure planning, design, and enhancement in the long run.

## Data Availability

We published the code used to extract the data on: https://github.com/EvacuationBehavior/Highway-Routing-Data-Processing. There are no restrictions to access and use/reuse the code.
